# The seasonal occurrence of periodontitis – a retrospective cohort study from a practice-based research network

**DOI:** 10.1007/s00784-024-05972-0

**Published:** 2024-10-14

**Authors:** Stefanie Anna Peikert, Stephanie Metzger, Anne Brigitte Kruse, Felix Mittelhamm, Eberhard Frisch, Kirstin Vach, Petra Ratka-Krüger, Johan Peter Woelber

**Affiliations:** 1 Department of Dental Medicine and Oral Health, Medical University of Graz, Billrothgasse 4, Graz, 8010 Austria; 2 Department of Operative Dentistry and Periodontology, Faculty of Medicine, University of Freiburg, Hugstetter Straße 55, 79106 Freiburg, Germany; 3 Private Dental Practice, Moorhof 7b, 22399 Hamburg, Germany; 4 Private Dental Practice, Industriestraße 17 A, 34369 Hofgeismar, Germany; 5 Institute of Medical Biometry and Statistics, Faculty of Medicine and Medical Center, University of Freiburg, Stefan-Meier-Straße 26, 79104 Freiburg im Breisgau, Germany; 6 Policlinic of Operative Dentistry, Periodontology, and Pediatric Dentistry, Medical Faculty Carl Gustav Carus, Technische Universität Dresden, Fetscherstraße 74, 01307 Dresden, Germany

**Keywords:** Seasonality, Periodontitis, Practice-based research network (PBRN)

## Abstract

**Objective:**

Many diseases are characterised by their seasonal appearance due to circannual changes in immune defence and a lifestyle that changes over the seasons. However, there is a lack of studies regarding the influence of seasonality on periodontitis. Therefore, the aim of this non-interventional, retrospective, observational study was to investigate the influence of seasonality on periodontitis.

**Materials and methods:**

Periodontal parameters of 5,908 patients of a practice-based research network (PBRN) were analysed. Probing pocket depth (PPD), Bleeding on Probing (BOP) and tooth mobility were assessed for seasonal fluctuations.

**Results:**

Within the PBRN patient data, seasonality was significantly associated with PPD of the individual months in patients with periodontitis. Pairwise comparison between the months showed significantly higher PPD in July compared to several months. PPD appeared significantly lower in December compared to June and November. Regarding season, the proportion of deep PPDs ($\:\ge\:$6 mm) was significantly higher in summer compared to autumn (*p* = 0.024). Concerning BOP, a significant difference between winter and autumn could be observed (*p* = 0.016). No seasonal influence was found for tooth mobility.

**Conclusion:**

This practice-network based study indicated a tendency for seasonal variations in periodontal parameters among periodontal patients. However, the differences did not achieve clinical relevance.

**Clinical relevance:**

Regarding the seasonality of several diseases there might also be an influence of seasons on the periodontium, which would have a potential influence on periodontal studies and daily periodontal examination.

**Supplementary Information:**

The online version contains supplementary material available at 10.1007/s00784-024-05972-0.

## Background

It has long been known that seasons influence the genesis and occurrence of many diseases [1]. There is evidence that physiological functions and conditions related to human disease have seasonal and circadian patterns, such as cardiovascular disease, autoimmune diseases, skin diseases, psychiatric disorders and mental illness [2–8]. Viral infections, such as influenza and even coronaviruses, also show a seasonal incidence pattern [9, 10]. These influences finally lead to a significant seasonality of mortality rates, which constantly show higher death rates in the winter season [11–13]. Seasonal differences may be caused by circannual changes in immune defence [14–16], as well as by a lifestyle that changes over the seasons, e.g. dietary habits or physical activity change [17–21]. These are influencing factors for various chronic inflammatory diseases, such as gingivitis [22] and periodontitis [23, 24].

Periodontitis as a biofilm-associated, inflammatory disease resulting in the loss of tooth-surrounding bone is closely associated with a lot of non-communicable chronic diseases like diabetes mellitus, obesity and metabolic syndrome [25–27]. This strong association between various oral diseases and these chronic diseases is a consequence of common risk factors, some of which have strong seasonal variations. In the context that diabetes is an important risk factor for periodontitis, Hermann et al., (2017) reported significantly higher values in their ten-year period study of HbA1c levels in patients with type 1 diabetes (T1DM) in the winter months and in June than in late summer [28]. Furthermore, Retnakaran et al., (2018) reported a higher prevalence of gestational diabetes (GDM) in the summer months and suspected a possible link between ambient temperature and blood glucose levels [29]. Seasonal patterns were also suspected concerning eating habits [30]. Moreover, it’s also known that dietary habits influence the inflammatory state of the periodontium [23, 31–33]. A further risk factor discussed for periodontal inflammation is the hormone vitamin D, whose serum concentration is strongly dependent on the season [34]. Also subject to seasonal fluctuations is melatonin [35–37]. It is involved in the regulation of the circadian light-dark cycle in the human body and is a powerful antioxidant and immunomodulator. It is also characterized by its anti-inflammatory ability, which may have a positive effect on periodontal parameters [38]. In summary, it has already been demonstrated in the literature that numerous risk factors for periodontitis are subject to seasonal fluctuations. However, little is known about the actual influence of the seasons on periodontal diseases.

Some studies showed a potential effect of seasonality on necrotizing ulcerative periodontal diseases [39–41]. For the summer months, for example, levels are about six times higher than in the spring and winter, and twice as high as in the fall. In addition, climatic conditions affect necrotising periodontal diseases (NPD), such as the occurrence of rainy season and drought [39, 42].

However, to the best of the author´s knowledge, there is a lack of studies regarding the influence of seasonality on periodontitis. This would be highly relevant not only for daily periodontal practice if the dental team could expect different clinical parameters in different months, but also for periodontal research, where study participants would react differently based on the season of examination.

Therefore, the aim of this non-interventional, retrospective, observational study was to investigate possible variations in prevalence across different seasons.

## Materials and methods

Periodontal patient data of this non-interventional, retrospective, observational study was based on a periodontal practice-based research network (PBRN) [43]. The PBRN consisted of graduates of the Master’s program in Periodontology and Implant Therapy at the University of Freiburg. As part of their education, they were trained according to the standard protocol of the recognized guidelines to ensure a similar level of treatment and training [44]. All of them routinely used a digital periodontal examination programme (ParoStatus, Parostatus.de GmbH, Germany) for documentation purposes in their practice, which made it possible to extract the periodontal findings from the practices and send them pseudonymized to the study center for further analysis.

In this study, 9 graduates of the master’s programme sent their treatment data to the study centre. Each participant was provided with information about the design and conduct of this non-interventional observational study and signed an informed consent form. Patients’ examination period of the different practices varied from October 1998 to April 2017, depending on when the ParoStatus program was implemented in each practice. The first examination which was recorded in the program was analysed. Due to the implementation of the new examination software ParoStatus, it is not possible to further distinguish whether this initial visit is from a patient undergoing primary periodontal treatment or supportive periodontal therapy. Just Patients with complete periodontal assessments were included, while those with incomplete or redundant data were excluded. After installation and extraction of the additional software, all patients who met the inclusion and exclusion criteria were compiled in a list. Individual files were generated for each of the measurement parameters probing pocket depth (PPD), Bleeding on Probing (BOP) and tooth mobility, which were saved in .csv format (comma-separated values or character-separated values). Via this software, clinical periodontal data of a total of 5908 patients was collected and underwent statistical analysis.

Within the scope of this retrospective observational study, the data material was evaluated to determine whether there was a seasonal influence on periodontitis.

To minimize the influence of periodontal therapy on a possible seasonality, only the periodontal parameters of the initial visit were used.

### Statistical procedures

When analysing the data of the PBRN, the mean PPD/BOP/tooth mobility value over all examined teeth at the first visit was calculated for each patient. Medians, mean values and standard deviations were calculated for a descriptive analysis of the data. Boxplots were used for a graphical presentation. Linear mixed regression models were used to analyse the influence of season or month on PPD, BOP and tooth mobility. For subsequent pairwise comparison Scheffé‘s method was used to correct for multiple testing. All analyses were performed with the statistical software STATA 16.1 (StataCorp LP, Lakeway Drive College Station, Texas, USA). *p* < 0.05 was considered statistically significant.

The seasons were defined by the following months:

- Spring = March, April, May.
- Summer = June, July, August.
- Autumn = September, October, November.
- Winter = December, January, February.

## Results

The demographic data are presented in Table 1.

**Table 1:** Demographic data

Parameter	
Examined patients	5908
Number of teeth	128,309
Average Age, years	56.53 ± 53.18
Recorded years	9.77
Average PPD	3.25±1.07
Average BOP	23±31

Average age of the patients in years (mean ± SD (standard deviation)), average recorded time in years, average PPD in mm (mean ± SD (standard deviation)), average BOP in % (mean ± SD (standard deviation))

Statistical analysis of the data sets revealed a significant seasonal influence on periodontitis about PPD and BOP. No seasonal influence was found with the investigated periodontal parameter tooth mobility.

### Probing pocket depth (PPD)

Table 2 shows the average PPD measured in millimetres for each month. The statistical analysis showed different values for the individual months (*p* = 0.0005). In the pairwise comparison between the months, July showed significantly higher PPD compared to January (contrast $\:\varDelta\:$ = 0.38; *p* = 0.020), February ($\:\varDelta\:$ = 0.35; *p* = 0.050), September ($\:\varDelta\:$ = -0.36; *p* = 0.047) and December ($\:\varDelta\:$ = -0.48; *p* = 0.001). Moreover, the month of December showed significantly lower PPD compared to June ($\:\varDelta\:$ = -0.42; *p* = 0.009), and November ($\:\varDelta\:$ = -0.41; *p* = 0.010). The corresponding Figure can be found in the supplement (supplemental material, Fig. 1). Concerning the seasons, the statistical analysis also showed different values (*p* < 0.001). Winter was found to show significantly lower values in comparison to all other seasons (spring ($\:\varDelta\:$ = -0.22 *p* < 0.001); summer ($\:\varDelta\:$ = -0.28 *p* < 0.001); autumn ($\:\varDelta\:$ = -0.14; *p* = 0.026)). Also, for autumn lower values than for summer were observed ($\:\varDelta\:$ = -0.15; *p* = 0.024). The corresponding table and figure can be found in the supplement (supplemental material, Table 1; Fig. 2).

**Table 2:** Average PPD measured in millimeters for each month. *N* = number of patients, median, mean and sd = standard deviation

month	*N*	probing depths (mm)		
		median	mean	sd
january	559	3.00	3.26	1.04
february	594	3.17	3.29	1.11
march	487	3.17	3.32	1.05
april	401	3.17	3.27	1.07
may	526	3.17	3.27	1.02
june	458	3.17	3.28	1.07
july	462	3.17	3.34	1.12
august	365	3.17	3.23	1.14
september	556	3.00	3.12	1.07
october	535	3.00	3.20	1.05
november	526	3.17	3.27	1.06
december	439	3.00	3.20	1.09
**total**	**5908**	**3.00**	**3.25**	**1.07**

Table 3 shows the distribution of the PPD per season in percentage in the categories < 4 mm, 4–6 mm and ≥ 6 mm [45]. Comparing the seasons with one another, autumn showed significantly lower values than summer for the $\:\ge\:$PPD 6 mm ($\:\varDelta\:$ = -1.25; *p* = 0.024).

**Table 3:** Percentage of the probing depths in different classes per season

probing depth		season			
		spring	summer	autumn	winter
4 mm		63.57	62.79	65.10	63.94
4–6 mm		29.71	29.60	28.55	29.15
$\:\ge\:$6 mm		6.72	7.61	6.35	6.91

### Bleeding on probing (BOP)

Table 4 shows the average BOP per month. Significant seasonal fluctuations in the BOP index were observed for the individual months (*p* = 0.011), after correction for multiple testing no significant differences in pairwise comparisons could be shown. The corresponding Figure can be found in the supplement (supplemental material, Fig. 3). Concerning the seasons, the statistical analysis also showed different values (*p* = 0.0068). Table 2; Fig. 4 of the supplemental material shows the distribution of the measured BOP values per season. Only a significant difference between winter and autumn could be observed ($\:\varDelta\:$ = -0.028; *p* = 0.016).

**Table 4:** Average BOP for each month. *N* = number of patients, mean and sd = standard deviation

month	*N*	Bleeding on Probing			
		mean		sd	
january	559	0.22		0.31	
february	594	0.22		0.30	
march	487	0.23		0.32	
april	401	0.23		0.31	
may	526	0.23		0.31	
june	458	0.24		0.31	
july	462	0.26		0.33	
august	365	0.21		0.30	
september	556	0.25		0.32	
october	535	0.25		0.32	
november	526	0.23		0.30	
december	439	0.21		0.30	
**total**	5908	0.23		0.31	

### Tooth mobility

Table 3 of the supplemental material shows the average tooth mobility observed in the corresponding month. No significant seasonal fluctuations could be detected (*p* = 0.952). In June the highest value (average mobility of 0.32) was observed.

No significance could be demonstrated concerning the average tooth mobility per season (*p* = 0.628). The corresponding table can also be found in the supplement (supplemental material, Table 4).

## Discussion

The aim of this non-interventional, retrospective, observational study was to investigate the influence of seasonality on periodontitis. For this reason, data of 5908 patients of a PBRN was analysed.

The results of the data analysis revealed a seasonal influence on periodontitis regarding PPD. However, the significant difference was in a minimal and clinically irrelevant range (< 0.5 mm of difference in PPD [46]).

No clinically relevant seasonal influence was found with the investigated periodontal parameter tooth mobility. Concerning BOP, a significant difference between winter and autumn could be observed (*p* = 0.016).

Periodontitis can be considered an expression of immune modulation, based on genetic and lifestyle factors (smoking, diabetes diet, stress, physical activity) [47]. Therefore, it is important to view all seasonal influences on the immune system. These immune functions and lifestyle factors are exposed to seasonal fluctuations. It was observed that lower temperatures lead to reduced production of proteins that can scavenge radicals, while day length affects lymphocyte activity, resulting in reduced immune function during the shorter days in winter compared to summer [48]. Another study demonstrated that reduced daily sun exposure is associated with reduced phagocytosis activity of granulocytes [49]. In this respect, a worsening of periodontal inflammation levels, especially in the winter months, would be expected. In addition, other diseases modulating the immune system, just like some risk factors of periodontitis, are also characterized by seasonal variations. For example, viral infections such as influenza or human coronaviruses (229E and OC43) are observed to be more prevalent during the winter months [9, 10]. Mortality rates, due to cardiovascular disease, vascular disease, and respiratory disease, were also higher in winter [50, 51]. Likewise, mental illnesses that have an impact on the immune system may be seasonal. Seasonal affective disorder (SAD) represents a typical seasonal form of depression with peaks in the winter months [3]. Finally, it should be noted that the development of periodontitis requires a comparatively long period. This could also be a reason why seasonality is rarely expressed in periodontitis. In this context, it is conceivable that periodontitis manifested in summer can still be diagnosed in winter. This would obscure the direct view of seasonality.

Concerning the number of patients, there were no significant fluctuations between the seasons. However, the lowest number of patients was consistently observed in August. In the study by Skach et al. (1970), August was also the month with the lowest incidence of ulcerative gingivitis [41]. This may probably due to August is a common holiday month. Because of the limiting nature of this study (retrospective cohort analysis), there is broad room for assumption as whether or not differences were found.

The significant by clinically irrelevant differences in PPD have to be discussed about modern living circumstances. In the Anthropocene era geographic and seasonal effects may be greatly levelled out. This includes modern food infrastructure throughout the year, with an almost constant food supply. Furthermore, nutrition is also a strong influencing factor of periodontal inflammation, among other factors [22, 32]. The increased off-season supply of imported products and their availability in supermarkets almost all year round contributes to reducing seasonal variations. This assumption is in line with a study carried out in Switzerland where food imports significantly reduced the seasonal fluctuations in nutrient intake, keeping it relatively stable throughout the year [30]. These findings certainly apply just as well to other wealthy industrial countries. Seasonal fluctuations in food availability are accordingly more observable in developing countries in particular [52]. There, the nutritional pattern seems to change even more, depending on the season, due to the cyclical availability of food [53]. Accordingly, it can be assumed that seasonally dependent periodontal changes could become noticeable in these countries, which would also have to be proven in future research.

About climate factors and geographic sun exposure it has to be noted, that the investigations of this study took place in German dental practices. On a global scale, however, certain differences due to different living conditions and seasonal weather and temperature fluctuations would be interesting to investigate. Moreover, one factor that contributes to the reduction of seasonality could be increased protection against extreme temperatures in households and working environments. Due to electricity and other modern methods, industrial populations (as in Germany) are largely independent of climatic factors. In winter, for example, people avoid cold by heating rooms, and in summer air conditioning systems protect against extreme heat. This means that temperature conditions can be kept largely constant throughout the year [54].

Besides, vitamin D supplements or the use of daylight lamps may help to maintain constant vitamin D levels in winter [55, 56]. In some countries, edible oils and fats are also enriched with vitamin D. These could be important sources of vitamin D, especially in winter when sunlight is rare. For example, the addition of vitamin D to margarine is compulsory in Norway, Denmark, the Netherlands, Belgium and Portugal and optional in Hungary, Switzerland, Spain, Italy, Portugal and Greece [56].

### Limitations

Retrospective cohort studies of PBRNs have several inherent limitations [43]. The investigated population has to be critically discussed. On the one hand, the data offered a view on a large and „real“ population of periodontal patients. On the other hand, the investigated population consisted of either initial periodontal treatment or supportive periodontal treatment. Thus, natural changes over the time due to a possible seasonality may be not that obvious. There were no available information about prior periodontal treatments in these patients. Accordingly, the presented data only applies for a population undergoing periodontal therapy.

Additionally, there is a certain bias in that the examinations are carried out by different practitioners who have all completed the Master’s program in Periodontology and Implant Therapy and have learned a treatment protocol according to the current guidelines, but there was no further calibration.

Furthermore, an additional population of healthy participants would offer a broader view on the research question.

A further limiting aspect is that no information on previous diseases and associated drug intake, body mass index (BMI), nor on dietary habits, oral hygiene, tobacco and alcohol consumption, physical activity, stress levels and vitamin D status, such as HbA1c levels and melatonin levels was transferred to the study centre. These seasonal confounding factors influence the severity of periodontal inflammation. As the patient data was all collected in Germany, geographically speaking this was a monocentric study. Moreover, the ParoStatus program does not collect several other potentially relevant data points that could have been included in the analysis. For instance, it does not provide information on the patient’s gender. Additionally, the program offers only limited insights into the severity of periodontitis, as it does not store radiological findings.

## Conclusion

In summary, the results of this retrospective data analysis of the practice-based patient cohort indicated a tendency for seasonal variations in periodontal parameters among periodontitis patients which, however, are of no clinical relevance. Accordingly, the month of an examination does not appear to have a considerable influence on the clinical parameters and the treatment outcome.

## Electronic supplementary material

Below is the link to the electronic supplementary material.

Supplementary Material 1

## Data Availability

No datasets were generated or analysed during the current study.

## Abbreviations

BMI: Body mass index
BOP: Bleeding on Probing
GDM: Gestational diabetes
PA: Periodontitis
PBRN: Practice-based research network
PPD: Probing pocket depths
SAD: Seasonal affective disorder
SD: Standard deviation
SPT: Supportive periodontal therapy
T1DM: Type 1 diabetes
