# Supplementary material for: The seasonal occurrence of periodontitis – a retrospective cohort study from a practice-based research network
Source: Clin Oral Investig. 2024 Oct 14;28(11):596. doi: 10.1007/s00784-024-05972-0 (PMC11473543; doi:10.1007/s00784-024-05972-0)
Supplement: Supplementary file 1 — Supplementary Material 1 [file 784_2024_5972_MOESM1_ESM.docx]

**Supplemental material**

Figure 1: Average PPD measured in millimeters for each month in box-plots.


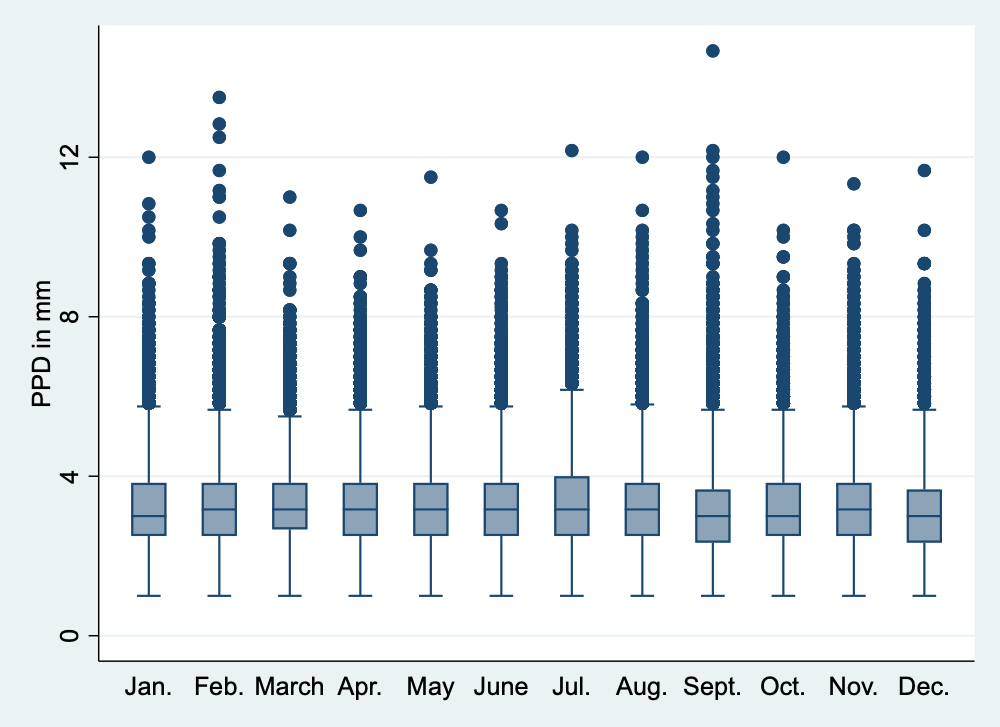


Figure 2: Average PPD measured in millimeters per season in box-plots.


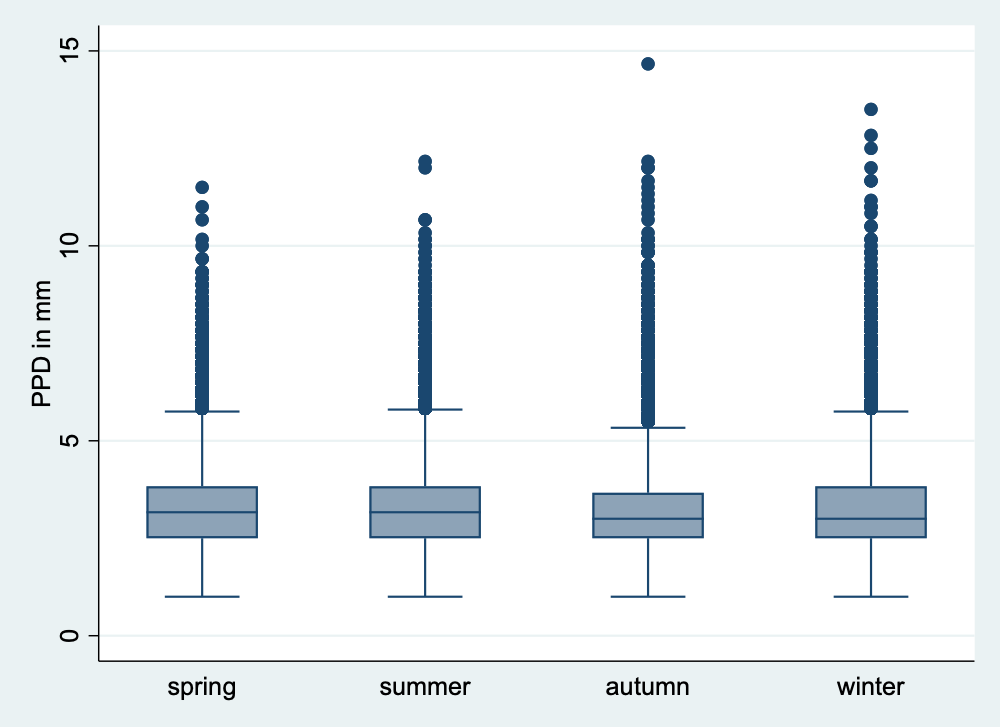


Figure 3: Average BOP per month in box-plots.


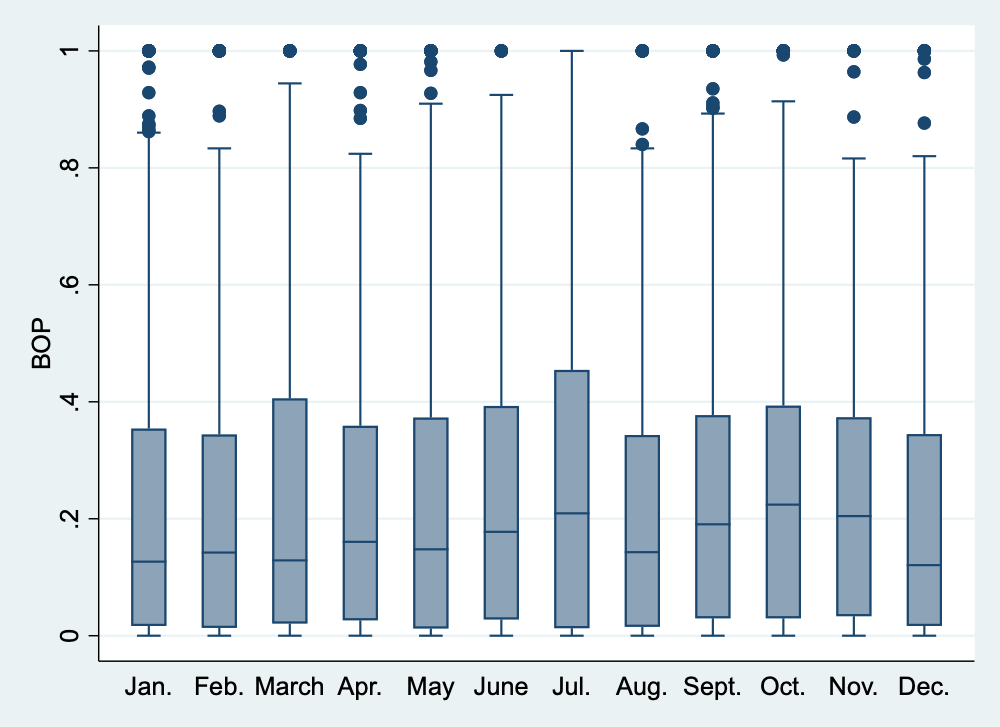


Figure 4: Average BOP per season in box-plots.

**
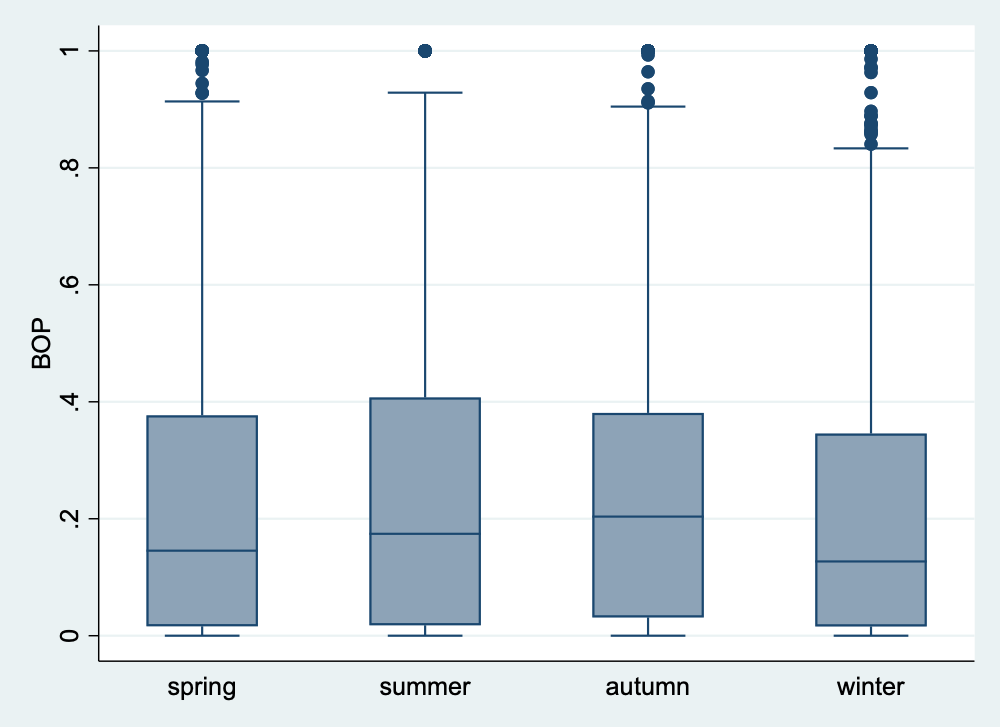
**

Table 1: Average PPD measured in millimeters per season. N = number of patients/teeth, median, mean and sd = standard deviation

| **season** | **N**  **patients** | **N**  **teeth** | **probing depths (mm)** | | |  |
| --- | --- | --- | --- | --- | --- | --- |
|  |  |  | **median** | **mean** | **sd** | |
| spring | 1414 | 30532 | 3.17 | 3.28 | 1.04 | |
| summer | 1285 | 28404 | 3.17 | 3.29 | 1.11 | |
| autumn | 1617 | 35823 | 3.00 | 3.19 | 1.06 | |
| winter | 1592 | 33550 | 3.00 | 3.25 | 1.08 | |

Table 2: Average BOP per season. N = number of patients, mean and sd = standard deviation

| **season** | **N** | **Bleeding on Probing** | |
| --- | --- | --- | --- |
|  |  | **mean** | **sd** |
| spring | 1414 | 0.23 | 0.31 |
| summer | 1285 | 0.24 | 0.31 |
| autumn | 1617 | 0.24 | 0.31 |
| winter | 1592 | 0.22 | 0.30 |

Table 3: Average tooth mobility for each month. N = number of patients, mean and sd = standard deviation

| **month** | **N** | **Tooth mobility** | |
| --- | --- | --- | --- |
|  |  | **mean** | **sd** |
| january | 559 | 0.12 | 0.33 |
| february | 594 | 0.11 | 0.33 |
| march | 487 | 0.10 | 0.32 |
| april | 401 | 0.10 | 0.31 |
| may | 526 | 0.10 | 0.32 |
| june | 458 | 0.32 | 0.36 |
| july | 462 | 0.12 | 0.35 |
| august | 365 | 0.10 | 0.32 |
| september | 556 | 0.11 | 0.31 |
| october | 535 | 0.10 | 0.30 |
| november | 526 | 0.11 | 0.33 |
| december | 439 | 0.11 | 0.36 |
| **total** | 5908 | 0.11 | 0.33 |

Table 4: Average tooth mobility per season. N = number of patients, mean and sd = standard deviation

| **season** | **N** | **Tooth mobility** | |
| --- | --- | --- | --- |
|  |  | **mean** | **sd** |
| spring | 1414 | 0.10 | 0.32 |
| summer | 1285 | 0.12 | 0.34 |
| autumn | 1617 | 0.11 | 0.32 |
| winter | 1592 | 0.11 | 0.34 |
